# NF-κB hyper-activation by HTLV-1 Tax induces cellular senescence, but can be alleviated by the viral anti-sense protein HBZ

**DOI:** 10.1186/1742-4690-8-S1-A200

**Published:** 2011-06-06

**Authors:** Huijun Zhi, Liangpeng Yang, Yu-Liang Kuo, Yik-Khuan Ho, Hsiu-Ming Shih, Chou-Zen Giam

**Affiliations:** 1 Department of Microbiology and Immunology Uniformed Services, University of the Health Sciences, Bethesda, MD, 20814, USA; 2 Institute of Biomedical Sciences, Academia Sinica, Taipei, 115, Taiwan

## 

Activation of I-κB kinases (IKKs) and NF-κB by the human T lymphotrpic virus type 1

(HTLV-1) trans-activator/oncoprotein, Tax, is thought to promote cell proliferation and transformation. Paradoxically, expression of Tax in most cells leads to drastic up-regulation of cyclin-dependent kinase inhibitors, p21CIP1/WAF1 and p27KIP1, which cause p53-/pRb-independent cellular senescence. Here we demonstrate that p21CIP1/WAF1-/p27KIP1-mediated senescence constitutes a checkpoint against IKK/NF-κB hyper-activation. Senescence induction by Tax is attenuated by mutations in Tax that reduce IKK/NF-κB activation and prevented by blocking NF-κB using a degradation-resistant mutant of I-κBα despite constitutive IKK activation. Small hairpin RNA-mediated knockdown indicates that RelA induces this senescence program by acting upstream of the anaphase promoting complex and RelB to stabilize p27KIP1 protein and p21CIP1/WAF1 mRNA respectively. Finally, we show that downregulation of NF-κB by the HTLV-1 anti-sense protein, HBZ, delay or prevent the onset of Tax-induced senescence. We propose that the balance between Tax and HBZ expression determines the outcome of HTLV-1 infection. Robust HTLV-1 replication and elevated Tax expression drive IKK/NF-κB hyperactivation and trigger senescence. HBZ, however, modulates Tax-mediated viral replication and NF-κB activation, thus allowing HTLV-1-infected cells to proliferate, persist, and evolve. Finally, inactivation of the senescence checkpoint can facilitate persistent NF-κB activation and leukemogenesis.

